# Neutrophil Extracellular Traps in Systemic Lupus Erythematosus: Pathogenic Mechanisms, Crosstalk with Oxidative Stress, and Antioxidant Therapeutic Potential

**DOI:** 10.3390/antiox15010025

**Published:** 2025-12-23

**Authors:** Xi Chen, Danni Gao, Matthew Wang, Lisheng Wang, Honghua Hu, Chengping Wen, Yujun Tang

**Affiliations:** 1 Jinhua Academy of Zhejiang Chinese Medical University, Jinhua 321000, China; chenxi@zhezhonglab.ac.cn (X.C.); gaodanni@zhezhonglab.ac.cn (D.G.); 202531124011089@zcmu.edu.cn (L.W.); 2 Faculty of Medicine and Health, University of New South Wales, Sydney, NSW 2052, Australia; z5358422@unsw.edu.au; 3 College of Pharmaceutical Sciences, Zhejiang University, Hangzhou 310058, China; honghuahu@zju.edu.cn; 4 Macquarie Medical School, Macquarie University, Sydney, NSW 2109, Australia; 5 Key Laboratory of Chinese Medicine Rheumatology of Zhejiang Province, College of Basic Medical Science, Zhejiang Chinese Medical University, Hangzhou 310053, China; 6 Innovative Research Center for Basic Medicine on Autoimmune Diseases, Ministry of Education, Hangzhou 310053, China; 7 Science and Technology Innovation Center, The Second Affiliated Hospital of Zhejiang Chinese Medical University, Hangzhou 310009, China

**Keywords:** neutrophil extracellular traps, systemic lupus erythematosus, oxidative stress, antioxidants, autoimmune diseases

## Abstract

Systemic lupus erythematosus (SLE) is a complex autoimmune disease characterized by autoantibody production and the formation of immune complexes (ICs), which lead to widespread inflammation and tissue damage. Neutrophil extracellular traps (NETs), web-like structures composed of DNA, histones, and antimicrobial proteins released by activated neutrophils, play a crucial role in innate immunity by defending against pathogens. However, excessive NET formation and ineffective clearance of these structures contribute to the development of SLE. This review explores the mechanisms behind NET formation in SLE, their relationship with oxidative stress, and the potential role of antioxidants in treatment. Research indicates that SLE patients exhibit two key abnormalities: excessive NET formation and impaired NET clearance. Excessive NET formation is driven by proinflammatory low-density granulocytes (LDGs) and immune complexes (ICs). Impaired NET clearance stems from reduced DNase1/DNase1L3 activity or anti-nuclease autoantibodies. These two abnormalities lead to elevated circulating NETs. These NETs act as autoantigen reservoirs, forming pathogenic NET–ICs that amplify autoimmune responses. Oxidative stress drives NET formation by activating NADPH oxidase. In contrast, various antioxidants, including enzymatic and non-enzymatic types, can inhibit NET formation via scavenging reactive oxygen species (ROS) and blocking NADPH oxidase activation. Preclinical studies show that antioxidants such as curcumin, resveratrol, and mitochondrial-targeted MitoQ reduce NET formation and ameliorate lupus nephritis; clinical trials confirm that curcumin and N-acetylcysteine (NAC) lower SLE disease activity and reduce proteinuria, supporting their role as safe adjuvant therapies. However, high-dose vitamin E may exacerbate autoimmunity, highlighting the need for dose optimization. Future research should aim to clarify the mechanisms underlying NET formation in SLE and to optimize new antioxidant therapies, including assessments of their long-term efficacy and safety.

## 1. Introduction

Systemic Lupus Erythematosus (SLE) is a chronic, multisystem autoimmune disease characterized by the production of autoantibodies and immune complexes (ICs) [1]. These can lead to inflammation and damage in multiple organs and tissues. SLE predominantly affects females, with a female-to-male ratio of approximately 9:1 [2,3,4]. It is more prevalent in individuals of African, Hispanic, and Asian descent than in those of European ancestry, with reported prevalence rates up to 3–5 times higher in some populations [5]. The clinical manifestations of SLE are highly variable, ranging from mild symptoms such as fatigue and joint pain to severe, life-threatening complications involving the kidneys, cardiovascular system, lungs, and central nervous system [6,7]. Despite significant advances in understanding SLE pathogenesis and the development of new therapeutic strategies, the disease remains challenging to diagnose and manage due to its heterogeneous clinical presentation and the complex interplay of genetic, environmental, and immunological factors.

Neutrophils, the most abundant type of white blood cells in humans, have long been recognized as the first line of defense against bacterial and fungal infections [8]. Traditionally, their role in immunity was understood primarily through phagocytosis, degranulation, and the production of reactive oxygen species (ROS) [9]. However, a groundbreaking discovery by Brinkmann et al. [10] revealed a novel mechanism by which neutrophils combat pathogens: the formation of neutrophil extracellular traps (NETs). NETs are web-like structures composed of extracellular DNA, histones, and antimicrobial proteins released by neutrophils to trap and kill pathogens [11]. This discovery reshaped our understanding of neutrophil biology, revealing their multifaceted roles beyond phagocytosis, including pathogen trapping and modulation of immune responses.

NET formation, termed “NETosis,” involves the release of chromatin from the neutrophil nucleus into the extracellular space [12]. This process is distinct from apoptosis and necrosis. It is regulated by a complex interplay of intracellular signaling pathways, including the nicotinamide adenine dinucleotide phosphate (NADPH) oxidase system and the translocation of neutrophil elastase (NE) and myeloperoxidase (MPO) from cytoplasmic granules to the nucleus [13,14]. The formation of NETs is triggered by various stimuli, including bacterial and fungal pathogens, as well as specific cytokines and ICs [15,16].

The discovery of NETs has not only enhanced our understanding of neutrophil-mediated antimicrobial defense but also revealed their involvement in a wide range of inflammatory and autoimmune diseases [17]. For instance, NETs have been implicated in the pathogenesis of SLE, where impaired NET degradation leads to the accumulation of autoantigens and immune system activation [18]. Similarly, in sepsis, excessive NET formation has been associated with thrombosis and multiple organ failure [19]. Furthermore, NETs have been shown to play a role in cancer progression, contributing to tumor growth, metastasis, and immune evasion [20].

Given the diverse roles of NETs in health and disease, there is a growing interest in understanding the mechanisms underlying NET formation and their impact on immune responses. This review aims to define the SLE-specific mechanisms of NET formation, including LDG-driven NETosis and impaired clearance, and to assess their contribution to autoantibody production and tissue damage. It also seeks to elucidate the bidirectional crosstalk between oxidative stress, such as NADPH oxidase and mitochondrial ROS, and NET formation in SLE pathogenesis. Furthermore, the review evaluates the therapeutic potential of antioxidants in SLE, including preclinical and clinical evidence for NET inhibition and symptom relief, and identifies gaps, such as the low bioavailability of curcumin and dose-dependent risks of vitamin E. Finally, it proposes future directions, such as developing targeted antioxidants, investigating NET subtype heterogeneity, and lytic versus non-lytic NETs in SLE.

## 2. Formation Mechanisms of NETs

### 2.1. Definition and Structure of NETs

NETs are specialized extracellular effector structures generated by activated neutrophils, defined as interconnected networks of DNA fibers decorated with a diverse array of granule-derived proteins and nuclear components, and evolved to entrap and eliminate invading microorganisms (e.g., bacteria, fungi, viruses) as a key component of innate host defense [21]. First identified by Brinkmann et al. [10] in 2004, NET formation primarily occurs via a lytic programmed cell death pathway. However, non-lytic release, preserving neutrophil membrane integrity and cellular viability, has also been observed under specific stimulatory conditions [22].

The structural core of NETs consists of both genomic DNA (derived from the neutrophil nucleus) and mitochondrial DNA (mtDNA) [23]. Notably, mtDNA within NETs exhibits enhanced immunostimulatory activity when oxidized during NET extrusion, a feature linked to amplified inflammatory responses in autoimmune contexts [24].

Embedded within this DNA scaffold are critical granule proteins that reinforce NET functionality, including MPO, NE, lysozyme, and matrix metalloproteinases (MMPs) [25]. These proteins not only contribute to microbial killing through enzymatic activity but also modulate tissue microenvironments by remodeling extracellular matrices [26].

Nuclear components, particularly histones, are another structural element of NETs, and their post-translational modifications mediated by enzymes such as peptidylarginine deiminase 4 (PAD4), play pivotal roles in shaping NET architecture and immunogenicity. PAD4 catalyzes the citrullination of arginine residues on histones, thereby disrupting electrostatic interactions between histones and DNA and promoting chromatin decondensation, a prerequisite for NET formation [27]. Additionally, ROS-dependent carbamylation of histones further alters their structure, increasing their pathogenic potential by enhancing autoantigenicity and tissue-damaging properties [28].

Collectively, the modular structure of NETs, integrating a flexible DNA backbone, functionally active granule proteins, and modified nuclear components, enables their dual roles in host protection and, when dysregulated, in driving chronic inflammation and tissue injury [29]. This structural complexity also underpins their involvement in systemic autoimmune and autoinflammatory diseases, where NET-derived components (e.g., citrullinated histones, oxidized mtDNA) act as autoantigens to perpetuate aberrant immune responses [30].

### 2.2. Formation Process of NETs

#### 2.2.1. ROS: Central Regulator of Chromatin Decondensation and NET Extrusion

ROS serve as a central regulator of NET formation, with their production and downstream effects tightly coupled to chromatin decondensation, protease activation, and extracellular trap extrusion [31]. As documented in the literature, ROS are primarily generated through two key pathways during NET induction: the membrane-bound NADPH oxidase complex (which drives the “respiratory burst”) and mitochondrial ROS release, both of which are essential for initiating and sustaining NET formation [32]. Upon stimulation by microorganisms, ICs, or autoantibodies, ROS first inhibits cytoskeletal polymerization. This disruption of the neutrophil cytoskeleton removes the physical barrier that restricts nuclear material movement, laying the foundation for subsequent chromatin extrusion [33]. Additionally, ROS mediate the intracellular release of critical proteases from cytoplasmic granules; these proteases then degrade histones and structural proteins like gasdermin D (GSDMD). This degradation reduces membrane stability and elevating cytoplasmic calcium (Ca^2+^) concentrations to further amplify chromatin decondensation signals [34]. Notably, ROS also contributes to post-translational modifications of NET components. For instance, ROS-driven oxidation of genomic and mtDNA within NETs not only impairs nuclease-mediated degradation of these nucleic acids but also enhances their recognition by innate immune sensors, such as the cGAS-STING pathway, linking ROS to both NET formation and subsequent immunostimulatory effects [35].

#### 2.2.2. NADPH Oxidase: Rate-Limiting Step in ROS Production and NETosis

As the primary source of ROS, activation of the NADPH oxidase complex is a rate-limiting step in NET formation [36]. This membrane-bound enzymatic complex is triggered by engagement of diverse neutrophil surface receptors, including cytokine receptors, Fcγ receptors (FcγRs), complement receptors, and damage-associated molecular pattern (DAMP) receptors [37]. Ligand binding to these receptors converges on one downstream signal: elevated intracellular Ca^2+^ levels. This signal activates protein kinase C (PKC), which then mediates the assembly of the NADPH oxidase complex at the cell membrane. Once assembled, the complex catalyzes the production of superoxide free radicals (the primary ROS in this pathway), a process known as the “respiratory burst” [38]. This burst of ROS not only activates downstream proteases (as noted earlier) but also promotes the fusion of neutrophil granules with the nucleus, a key event that delivers granule proteins (e.g., MPO, elastase) to chromatin [39]. Importantly, NADPH oxidase activity is not confined to the plasma membrane; mitochondrial ROS production, which can be synergistically activated by NADPH oxidase-derived ROS, further reinforces NET formation by sustaining oxidative stress and amplifying chromatin decondensation [40]. Disruption of NADPH oxidase function, either via genetic mutations or pharmacological inhibition, has been shown to abrogate NET formation in both murine models and human neutrophils, confirming its non-redundant role in this process [41].

#### 2.2.3. MPO and NE: Synergistic Drivers of Chromatin Remodeling

MPO and NE, two abundant granule proteins in neutrophils, play complementary and indispensable roles in NET formation by modulating chromatin structure and degrading key cellular components [42]. NE, a serine protease, is among the first granule proteins activated by ROS (via NADPH oxidase signaling); once released into the cytoplasm, it translocates to the nucleus, where it degrades core histones (e.g., H3, H4) and nuclear structural proteins. This degradation disrupts the compact organization of chromatin, initiating the decondensation process essential for NET extrusion [43]. Elastase also targets GSDMD, a pore-forming protein at the nuclear and plasma membranes; cleavage of GSDMD by elastase forms membrane pores, increasing membrane permeability, elevating cytoplasmic Ca^2+^ levels, and further facilitating the release of decondensed chromatin [44]. MPO, another granule-derived enzyme, contributes to NET formation through both enzymatic and oxidative mechanisms: it catalyzes the production of hypochlorous acid (HOCl) from hydrogen peroxide (H_2_O_2_), and this highly reactive oxidant enhances the degradation of histones and structural proteins by elastase, acting as a “co-factor” to amplify elastase-mediated chromatin remodeling [45]. Beyond its enzymatic role, MPO also participates in post-translational modifications of NET components. It mediates the carbamylation of histones (via ROS-dependent reactions), which disrupt histone–DNA interactions, promoting chromatin decondensation and increasing histone immunogenicity, making them more likely to act as autoantigens in autoimmune diseases [46]. Together, MPO and elastase synergistically promote efficient NET formation: MPO-derived oxidants prime chromatin by enhancing elastase’s ability to degrade histones; in turn, elastase cleaves nuclear structural proteins to disrupt membrane integrity, enabling MPO and other granule proteins to bind the NET DNA scaffold. This synergistic interaction ensures efficient NET formation and modulates NET pathogenicity [47]. This collaboration not only facilitates the mechanical release of DNA but also shapes the composition and pathogenic potential of NETs, linking their activity to both microbial clearance and, in dysregulated states, to tissue damage and autoimmunity [48].

### 2.3. Physiological Functions of NETs

NETs exert critical physiological functions closely tied to host homeostasis, and their role in host defense is among the best-characterized and evolutionarily conserved [49]. Structurally composed of extracellular DNA fibers adorned with granule-derived proteins, including MPO, NE, and lysozyme. NETs act as both physical and enzymatic barriers, ensnaring a broad spectrum of invading microorganisms, including bacteria, fungi, and viruses [50]. Upon encountering microbial pathogens or their products (e.g., lipopolysaccharides, viral proteins), activated neutrophils deploy NETs to trap these invaders within the DNA mesh, preventing their dissemination to distant tissues; this physical sequestration is complemented by the enzymatic activity of granule proteins embedded in the NET scaffold, which directly degrade microbial cell walls, proteins, and nucleic acids to eliminate the pathogen [51]. For instance, MPO catalyzes the production of reactive oxidants that disrupt microbial membrane integrity, while NE cleaves key microbial virulence factors, thereby synergistically enhancing killing efficacy [52]. Notably, NET formation can occur via both lytic and non-lytic pathways. The latter preserves neutrophil viability, allowing the same cell to contribute to sustained host defense through repeated NET release or phagocytosis [53]. This dual capacity for physical trapping and enzymatic clearance positions NETs as a pivotal component of the innate immune response, bridging the gap between immediate pathogen containment and subsequent adaptive immune activation [54].

Beyond their role in direct pathogen elimination, NETs also function as central modulators of inflammatory responses, orchestrating the activation and recruitment of other immune cells to fine-tune the body’s response to threats [55]. A key mechanism underlying this modulation is the ability of NET components to engage innate immune sensors on cells such as macrophages and plasmacytoid dendritic cells (pDCs). For example, NET-derived double-stranded DNA (both genomic and oxidized mtDNA) is recognized by the cGAS-STING pathway in macrophages, triggering the production of type I interferons (IFNs), a family of cytokines that amplify antiviral immunity and prime adaptive immune responses [56]. Additionally, NETs are enriched with immunostimulatory molecules, including interleukin-33 (IL-33), high-mobility group box 1 (HMGB1), and the antimicrobial peptide cathelicidin LL-37; these molecules bind to receptors on immune cells to promote their maturation, enhance antigen presentation, and stimulate the secretion of pro-inflammatory cytokines [57]. NETs drive inflammatory amplification through two key mechanisms. First, they activate the complement system by facilitating the binding of complement component 1q (C1q) to NET components. Second, they recruit additional neutrophils and monocytes to sites of injury or infection. Together, these actions create a positive feedback loop that reinforces the inflammatory response [58]. Importantly, NETs are not solely pro-inflammatory: under specific conditions, aggregated NETs can exert anti-inflammatory effects by forming stable meshes that degrade pro-inflammatory cytokines and chemokines via embedded proteases, thereby limiting excessive immune cell infiltration and promoting the resolution of inflammation. This essential balance prevents tissue damage while ensuring effective pathogen clearance [59].

## 3. Role of NETs in SLE

### 3.1. Immunopathological Mechanisms of SLE

In SLE, the production of autoantibodies arises from a multifaceted breakdown of immune tolerance, driven by intrinsic B cell dysregulation and extrinsic microenvironmental cues [60]. Beyond the well-characterized antinuclear antibodies (ANA) and anti-double-stranded DNA (anti-dsDNA) antibodies—where ANA serves as a sensitive yet nonspecific diagnostic marker, and anti-dsDNA correlates with lupus nephritis activity—SLE patients also generate autoantibodies targeting ribonucleoprotein complexes, such as anti-Smith (anti-Sm), anti-Ro antibody/anti-Sjögren’s Syndrome A antibody (anti-Ro/SSA), and anti-La antibody/anti-Sjögren’s Syndrome B antibody (anti-La/SSB) [61]. Anti-Sm antibodies exhibit over 90% specificity for SLE, making them a definitive diagnostic tool, while anti-Ro/SSA antibodies are associated with cutaneous manifestations and neonatal lupus [62]. The cellular mechanisms underlying this autoantibody production are tightly linked to B-cell tolerance defects: genetic variants, including loss-of-function mutations in protein kinase C delta (PRKCD) and gain-of-function mutations in Toll-like receptor 7 (TLR7), enable the survival and proliferation of autoreactive B cells [63]. Notably, escape from X-chromosome inactivation of TLR7 in some female patients further amplifies B-cell hyperactivation [64]. Additionally, aberrant extrafollicular B cell responses play a pivotal role: CD11c^+^T-bet^+^ age-associated B cells (ABCs), a proinflammatory B cell subset, are significantly expanded in the peripheral blood of patients with SLE [65]. These ABCs differentiate into plasmablasts under the stimulation of interleukin-21 (IL-21) secreted by peripheral helper cells (Tph), contributing to sustained autoantibody production [66]. NETs further fuel this process by acting as a reservoir of modified autoantigens—such as citrullinated histones and oxidized mtDNA—which not only directly engage autoreactive B cells but also activate pDCs to secrete type I IFNs, creating a feedforward loop that reinforces B cell activation [67]. Defects in nucleic acid clearance, including reduced activity of Deoxyribonuclease 1-like 3 (DNase1L3), prolong extracellular DNA persistence, providing a continuous source of autoantigens for antibody generation [68].

ICs, formed by the binding of autoantibodies to self-antigens, are central pathogenic mediators in SLE, driving tissue damage through complement activation, inflammatory cell recruitment, and direct tissue deposition [69]. The composition of these ICs is heterogeneous: they may include anti-dsDNA antibodies bound to NET-derived genomic DNA or nucleosomes, anti-Ro/SSA antibodies complexed with cytoplasmic RNA, or autoantibody-coated microparticles released from apoptotic cells—these microparticle-associated ICs exhibit enhanced tissue adhesion and complement-activating capacity [70]. A critical pathogenic step is impaired clearance of ICs: SLE patients often show deficiencies in early complement components (e.g., C1q, C4), which are essential for opsonizing ICs and facilitating their phagocytosis by macrophages [71]. Moreover, autoantibodies targeting DNase1 or DNase1L3 inhibit DNA degradation within ICs, preventing their dissolution and promoting their accumulation in the circulation [72]. Once formed and retained, ICs exert their pathogenic effects through two primary pathways: first, they activate the classical complement cascade, generating anaphylatoxins that recruit neutrophils and monocytes to sites of inflammation and enhance their proinflammatory activity; second, they engage FcγRs on innate immune cells. pDCs internalize ICs and trigger type I IFN production via TLR7/9 sensing of nucleic acids, while macrophages release tumor necrosis factor-α (TNF-α) and interleukin-1β (IL-1β) upon IC binding, amplifying local inflammation [73]. In target organs such as the kidney, ICs deposit in the glomerular basement membrane and mesangium, disrupting filtration function and inducing podocyte injury; in the skin, IC deposition at the dermal–epidermal junction contributes to erythematous lesions [74]. This cycle of IC formation, impaired clearance, and inflammatory activation underpins the chronic tissue damage observed in SLE [75].

The pathogenesis of SLE is further perpetuated by the activation of multiple inflammatory cell types, including neutrophils, macrophages, and dendritic cells (DCs), which release proinflammatory cytokines and mediate tissue destruction [76]. Neutrophils exhibit distinct functional abnormalities in SLE: low-density granulocytes (LDGs), a proinflammatory subset, are expanded in peripheral blood and exhibit an increased propensity for NETosis [77]. These NETs are enriched in oxidized mtDNA, which activates the cGAS-STING pathway in macrophages and DCs, driving type I IFN production. Additionally, SLE neutrophils are prone to ferroptosis—an iron-dependent form of cell death characterized by lipid peroxidation—releasing DAMPs such as HMGB1 and heat shock protein 60 (HSP60) that further activate innate immune responses [78]. Macrophages undergo M1 polarization in SLE, a phenotype associated with enhanced secretion of TNF-α and IL-1β. M1 macrophages accumulate in inflamed tissues (e.g., renal interstitium, cutaneous lesions) and contribute to tissue injury by phagocytosing ICs and releasing MMPs that degrade the extracellular matrix. DCs, particularly pDCs and conventional dendritic cells (cDCs), play specialized roles: pDCs are the primary source of type I IFNs in SLE, activated by IC-derived nucleic acids via TLR7/9, and their secreted IFNs reinforce B cell activation and T cell differentiation; cDCs, upon uptake of autoantigens (e.g., nucleosomes), present these antigens to CD4^+^ T cells, promoting their differentiation into T follicular helper cells (Tfh) or Th17 cells [79]. Tfh cells secrete IL-21 to support B cell plasmablast differentiation, while Th17 cells produce interleukin-17 (IL-17) to exacerbate synovial inflammation and renal injury [80]. Importantly, the cytokines released by these activated cells (e.g., type I IFNs, TNF-α) create a proinflammatory microenvironment that further activates neighboring immune cells, establishing a positive feedback loop that sustains chronic inflammation and multi-organ damage in SLE [81]. In addition to excessive NET formation, impaired clearance of these structures further exacerbates SLE pathogenesis—creating a cumulative pool of extracellular autoantigens that fuel autoimmune responses.

### 3.2. Abnormalities of NETs in SLE: Production, Clearance, and Autoantigen Function

#### 3.2.1. Excessive NET Formation: LDG-Driven Pathogenesis

Patients with SLE exhibit expanded populations of proinflammatory LDGs. These LDGs undergo spontaneous NETosis due to mitochondrial ROS overproduction and PAD4-mediated histone citrullination. SLE-derived ICs and autoantibodies further trigger NET formation via FcγRIIA/TLR engagement, even in healthy neutrophils exposed to SLE serum [82]. This heightened NETosis is not uniform across all neutrophil subsets. LDGs, a proinflammatory subset expanded in SLE peripheral blood, display a markedly increased propensity for spontaneous NET formation compared to normal-density neutrophils (HDNs) in both patients with SLE and healthy controls [83]. LDGs in SLE are driven toward NETosis by multiple pathogenic cues, including overproduction of mitochondrial reactive oxygen species (mtROS) [84]. Oxidized mtDNA released during this process not only constitutes a key component of NETs but also enhances their immunostimulatory capacity by activating the cGAS-STING pathway in myeloid cells [85]. Additionally, SLE-derived ICs and autoantibodies directly trigger NET formation via engagement of FcγRIIA or Toll-like receptors on neutrophils [86]. Notably, even neutrophils from healthy individuals, when exposed to SLE serum or purified SLE autoantibodies, exhibit increased NET production, indicating that soluble factors in the SLE microenvironment prime neutrophils for NETosis [87]. The enzyme PAD4, which mediates histone citrullination and chromatin decondensation, also plays a pivotal role in SLE-associated excessive NETosis: PAD4 inhibition in murine lupus models reduces NET formation, autoantibody levels, and renal injury, though conflicting data exist regarding its protective vs. pathogenic role in specific contexts [88]. Collectively, this dysregulated NET production, driven by abnormal neutrophil subsets, proinflammatory stimuli, and altered intracellular signaling, creates a sustained pool of extracellular autoantigens that fuels SLE pathogenesis [89].

#### 3.2.2. Impaired NET Clearance: DNase and Complement Defects

Compounding the issue of excessive NET formation, patients with SLE exhibit profound impairment in NET clearance, a defect that perpetuates NET accumulation in tissues and the circulation and exacerbates autoimmune pathology [90]. Clearance of NETs is compromised by: (1) autoantibodies targeting DNase1/DNase1L3; (2) genetic mutations in DNase1L3; and (3) C1q-mediated inhibition of DNase1 activity. Reduced DNase activity correlates with higher circulating NET remnants (MPO-DNA complexes) and disease activity, particularly in lupus nephritis [91,92].

The primary mediators of NET degradation are DNase enzymes, particularly DNase1 and DNase1L3, whose activity is frequently compromised in SLE [91]. This impairment arises through three key mechanisms: (1) autoantibodies targeting DNase1 or DNase1L3 (e.g., anti-DNase1L3 IgG) in ~30–40% of patients with SLE, which directly neutralize nuclease activity [93]; (2) genetic variants or loss-of-function mutations in DNase1/DNase1L3 (e.g., homozygous DNase1L3 null mutations) linked to early-onset SLE [94]; and (3) complement C1q binding to NET DNA, which inhibits DNase1 activity [92]. Clinically, impaired NET clearance correlates with disease activity: Lupus nephritis patients exhibit lower DNase1L3 activity compared to patients with SLE without renal involvement, and reduced DNase activity is associated with higher levels of circulating NET remnants (e.g., MPO-DNA complexes), anti-dsDNA autoantibodies, and hypocomplementemia [95]. This failure to clear NETs not only prolongs exposure to autoantigens but also promotes the formation of stable NET-autoantibody complexes that resist degradation [96].

#### 3.2.3. NETs as Autoantigen Reservoirs: NET–IC Formation

NETs serve as a critical “autoantigen reservoir” in SLE, as their components—including genomic DNA, oxidized mtDNA, citrullinated histones, and granule proteins—directly interact with autoantibodies to form pathogenic ICs [97]. These NET-derived ICs exhibit unique immunostimulatory properties: for example, complexes of NET DNA (oxidized mtDNA or genomic DNA) with anti-dsDNA antibodies or the antimicrobial peptide LL37 activate pDCs via Toll-like receptor 9 (TLR9), triggering robust production and secretion of type I IFNs, a hallmark of SLE pathogenesis [98]. Similarly, ICs composed of citrullinated histones and anti-citrullinated histone H3 (CitH3) antibodies engage B cells via B cell receptors (BCRs) and TLRs, driving B cell differentiation into autoantibody-secreting plasma cells and amplifying the autoimmune response [99]. NET-ICs also activate neutrophils and monocytes via FcγRIIA, inducing further NETosis and secretion of proinflammatory cytokines, creating a self-perpetuating “NET-IC-inflammatory loop” [100]. Significantly, these ICs deposit in target organs, such as the kidney: in lupus nephritis, NET-ICs accumulate in glomeruli, where they activate the complement system (via the classical pathway) and recruit inflammatory cells, leading to podocyte injury, mesangial proliferation, and renal fibrosis [101]. Additionally, NET components, such as NE and MPO, within these ICs degrade extracellular matrix proteins, exacerbating tissue damage [102]. Together, the interactions between NETs and autoantibodies are central to SLE progression, linking innate immune dysregulation to adaptive autoimmunity and end-organ injury [103].

### 3.3. Pathological Roles of NETs in SLE

**Figure 1:**
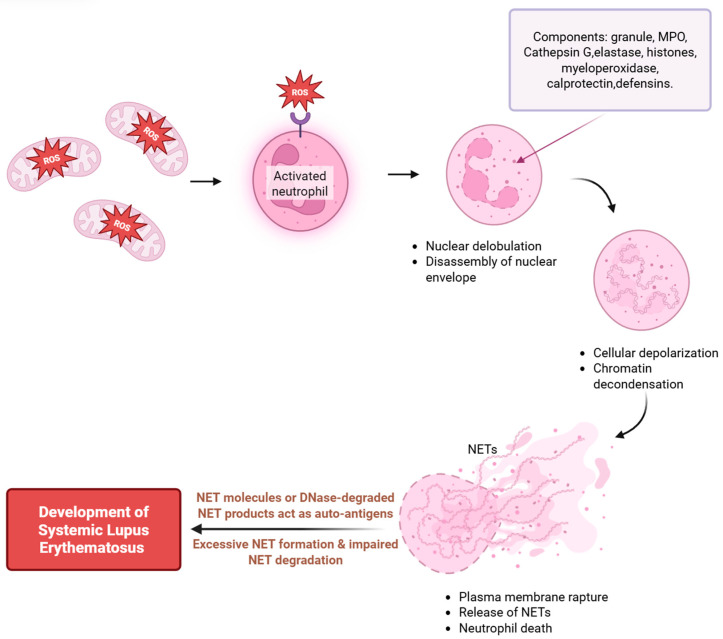
The formation mechanism of NETs in SLE.

NETs play a pivotal role as a central pathological mediator in SLE by orchestrating multifaceted proinflammatory effects that perpetuate autoimmune activation and tissue damage [104] (Figure 1). A key mechanism by which NETs amplify inflammatory responses is their ability to act as a “danger signal reservoir,” activating a spectrum of innate and adaptive immune cells. For instance, NET-derived components—including oxidized mtDNA, CitH3, and HMGB1—engage pattern recognition receptors (PRRs) on pDCs, such as TLR9 and cGAS-STING [105]. This activation triggers robust secretion of type I IFNs, a hallmark of SLE pathogenesis that further primes neutrophils for NETosis and drives B cell differentiation into autoantibody-secreting plasma cells [106]. Macrophages, upon uptake of NET fragments via FcγRIIA, undergo M1 polarization and release proinflammatory cytokines, including TNF-α and IL-1β, which recruit additional neutrophils and monocytes to inflamed sites [107]. Moreover, NETs provide a scaffold for the formation of ICs with autoantibodies, and these ICs further activate neutrophils and B cells via FcγR and BCR cross-linking, creating a self-perpetuating inflammatory loop [108]. Notably, LDGs—an aberrant neutrophil subset expanded in SLE—synergize with NETs to enhance inflammation: LDGs not only exhibit increased spontaneous NET formation but also secrete higher levels of IL-8. This chemokine amplifies neutrophil recruitment and activates endothelial cells, further exacerbating the proinflammatory microenvironment [109].

Beyond amplifying inflammation, NETs directly mediate tissue damage in SLE through their cytotoxic components and by modulating cellular homeostasis in target organs [110]. The cationic histones within NETs exert direct cytotoxicity on vascular endothelial cells by disrupting membrane integrity, thereby increasing permeability and causing endothelial dysfunction [111]. NET-associated enzymes exacerbate this: NE degrades extracellular matrix proteins, while MPO generates HOCl to oxidize endothelial proteins, thereby impairing barrier function [112]. In the kidney—a significant target of SLE—NETs deposit in glomeruli, where NE and MPO degrade podocyte proteins (e.g., nephrin), thereby disrupting the glomerular filtration barrier and contributing to proteinuria [113]. Additionally, NET-derived MMP-9 activates endothelial MMP-2, promoting endothelial-to-mesenchymal transition (EndMT) of glomerular endothelial cells—a process linked to renal fibrosis and progressive lupus nephritis [114]. In the lungs, NETs disrupt the microvascular endothelial barrier, facilitating diffuse alveolar hemorrhage (DAH) in severe SLE; here, NE and cathepsin G cleave lung epithelial cell junctions, while NET-induced IL-8 recruitment of neutrophils amplifies tissue destruction [115]. Collectively, these effects underscore NETs as direct effectors of tissue injury across multiple organ systems in SLE.

NETs also contribute to SLE-associated thrombotic complications by promoting platelet activation, activating the coagulation cascade, and stabilizing thrombi [116]. NETs bridge inflammation and thrombosis via three key mechanisms. First platelet activation occurs as histones bind to platelet TLR2 and TLR4, while NE and cathepsin G activate platelet protease-activated receptor 4 (PAR4), inducing granule release and integrin αIIbβ3 activation [116,117]. Second, coagulation cascade activation occurs because NETs express tissue factor, the initiator of the extrinsic pathway, and NE and cathepsin G degrade anticoagulants such as tissue factor pathway inhibitor (TFPI) and antithrombin III, thereby shifting the balance toward a prothrombotic state [117]. Third, thrombus stabilization occurs as the NET DNA scaffold concentrates platelet fibrinogen and von Willebrand factor, thereby enhancing platelet aggregation and fibrin deposition [118]. Clinically elevated levels of CitH3 or MPO-DNA complexes correlate with increased risk of cardiovascular disease and pulmonary embolism in SLE [116]. Notably, in secondary antiphospholipid syndrome (APS), a common thrombotic complication of SLE, NETs exert unique pathogenic effects driven by anti-β2-glycoprotein I (anti-β2-GPI) antibodies. A recent study demonstrated that anti-β2-GPI antibodies from SLE-APS patients can directly induce NET formation in healthy neutrophils and exhibit 93.6% colocalization with NE, without inhibiting DNase I-mediated NET degradation [119]. These anti-β2-GPI-induced NETs have a distinct proteomic profile and specifically amplify endothelial cell activation (upregulating TF, VCAM-1, and ICAM-1), thereby exacerbating thrombotic risk in SLE-APS [119]. Thus, NETs bridge inflammation and thrombosis in SLE, representing a critical link between autoimmune activation and organ damage.

## 4. Role of Oxidative Stress in NET Formation and SLE

### 4.1. Definition and Mechanisms of Oxidative Stress

Oxidative stress is a critical pathophysiological condition in SLE, characterized by a disrupted balance between reactive species production and the capacity of the antioxidant defense system, favoring excessive accumulation of reactive species [120]. At the core of oxidative stress are ROS and reactive nitrogen species (RNS), highly reactive molecules that can damage cellular components [121]. Under normal physiological conditions, ROS and RNS play essential roles in various cellular signaling pathways and regulatory processes [122]. In SLE, however, their production often exceeds the capacity of antioxidant defense mechanisms, leading to chronic oxidative stress [123].

The primary sources of ROS include the mitochondrial electron transport chain, cytochrome P450 enzymes, NADPH oxidases, and xanthine oxidase [124]. In eukaryotic cells, over 90% of ROS are generated by mitochondria, where electrons escaping from the electron transport chain interact with molecular oxygen to form superoxide anion radicals (••O_2_^−^) [125]. These radicals can also be converted into other ROS, such as H_2_O_2_ and hydroxyl radicals (•OH) [126]. RNS, on the other hand, are mainly derived from nitric oxide (NO) and its derivatives, such as peroxynitrite (ONOO^−^) [127]. While these reactive species have critical physiological functions at low concentrations, their excessive accumulation in SLE can lead to significant oxidative damage [128].

Oxidative stress exerts detrimental effects on various cellular components, including lipids, proteins, and DNA [129]. Lipid peroxidation, a common consequence of oxidative stress, involves the oxidation of polyunsaturated fatty acids in cell membranes, leading to the formation of toxic aldehydes such as malondialdehyde (MDA) and 4-hydroxynonenal (4-HNE) [130]. These aldehydes can disrupt membrane integrity and fluidity, impairing cellular functions [131]. Protein oxidation results in carbonylation, modification of amino acid residues, and protein cross-linking, which can alter protein structure and function, leading to enzyme inactivation and impaired cellular processes [132]. DNA damage caused by oxidative stress includes base modifications, strand breaks, and cross-linking, which can interfere with DNA replication and transcription, potentially leading to mutations and genomic instability [133]. Moreover, oxidative stress can activate various intracellular signaling pathways, such as the mitogen-activated protein kinase (MAPK) and nuclear factor-kappa B (NF-κB) pathways, further exacerbating cellular inflammation and apoptosis in SLE [134].

In summary, oxidative stress, characterized by an imbalance between ROS and RNS production and the capacity of antioxidant defense, can cause extensive damage to cellular components and contribute to the pathogenesis of SLE [135]. Understanding the mechanisms underlying oxidative stress and its regulation in SLE is crucial for elucidating the disease’s etiology and developing effective therapeutic strategies [136].

### 4.2. Role of Oxidative Stress in NET Formation: General Mechanisms and SLE-Specific Drivers

#### 4.2.1. General Mechanisms: ROS as a NETosis Trigger

Oxidative stress, particularly ROS generation, plays a pivotal role in NET formation [137]. The activation of the NADPH oxidase complex is a crucial step in ROS production, which subsequently leads to the release of DNA and histones, culminating in NET formation [138]. For instance, Ermert et al. [139] demonstrated that phorbol 12-myristate 13-acetate (PMA) activates the NADPH oxidase complex in neutrophils, resulting in ROS production and NET formation. Similarly, Hakkim et al. [137] confirmed the essential role of NADPH oxidase in NET formation, highlighting that ROS are indispensable for this process.

Conversely, antioxidants have been shown to inhibit NET formation by scavenging ROS [140]. Antioxidants such as superoxide dismutase (SOD), catalase, and glutathione (GSH) can effectively neutralize ROS, thereby reducing NET formation [141]. Fuchs et al. [142] reported that antioxidants like thiourea, dimethylthiourea, and N-acetylcysteine (NAC) significantly attenuate PMA-induced NET formation. Additionally, Dömer et al. [104] revealed the importance of mitochondrial ROS in Ca^2+^ ionophore-induced NET formation and demonstrated that mitochondrial ROS scavengers can inhibit NET formation.

Moreover, the role of glutathione reductase (Gsr) in NET formation has been elucidated [143]. Yan et al. [144] found that Gsr-deficient neutrophils exhibit impaired NET formation under oxidative stress, indicating that Gsr is crucial for maintaining neutrophil antioxidant capacity and supporting NET formation.

In summary, ROS are central to NET formation, while antioxidants inhibit this process by neutralizing ROS [140]. These findings underscore the significance of oxidative stress in NET formation and provide a foundation for developing therapeutic strategies targeting NET-related diseases [145].

#### 4.2.2. SLE-Specific Oxidative Stress Amplification

In SLE, oxidative stress exacerbates NET formation. Three key mechanisms primarily drive this process.

Firstly, LDGs exhibit a marked increase in mitochondrial ROS production. This heightened ROS generation drives spontaneous NETosis, leading to the release of oxidized mtDNA. This mtDNA, a potent immunostimulatory component of NETs, activates the cGAS-STING pathway in myeloid cells, further fueling inflammation and immune responses [84,88].

Secondly, patients with SLE often suffer from impaired antioxidant defenses. Reduced serum levels of antioxidants such as vitamin E and β-carotene, coupled with lower enzymatic antioxidant activities, result in increased ROS accumulation. This imbalance further exacerbates oxidative stress and promotes NET formation [144].

Thirdly, a self-sustaining feedback loop between NETs and ICs amplifies oxidative stress. Pathogenic NET–ICs activate neutrophils via FcγRIIA, which boosts NADPH oxidase activity and ROS production. This creates a vicious cycle: oxidative stress and NET formation reinforce one another, leading to chronic inflammation and tissue damage in SLE [108].

#### 4.2.3. ROS Species-Specific Regulation of NET Formation Pathways and Correlation with SLE Phenotypes

Oxidative stress in SLE is not a uniform process. ROS and RNS selectively activate NET formation pathways, which directly correlate with the heterogeneity of SLE clinical phenotypes. This species-specific interaction explains why oxidative stress-driven NETosis manifests differently across patients, as detailed below:

MtROS: PAD4-Dependent NETosis and Lupus Nephritis

MitROS, primarily generated by dysfunctional mitochondria in LDGs, is a key driver of PAD4-mediated histone citrullination, a rate-limiting step for chromatin decondensation and NET extrusion [84,87,146]. Unlike other ROS, mtROS directly accumulate in the neutrophil nucleus, where they activate PAD4 by oxidizing its cysteine residues [146]. The resulting CitH3-enriched NETs are highly immunogenic: they bind anti-dsDNA antibodies to form NET-ICs that preferentially deposit in the glomerular basement membrane of the kidney [147]. Clinically, elevated mtROS levels in LDGs correlate with increased proteinuria and renal histological activity in patients with lupus nephritis. Preclinical studies further validate this link: mitochondrial-targeted antioxidants reduce mtROS production, inhibit PAD4 activity, and ameliorate renal injury in MRL/lpr mice [147], confirming the mtROS-PAD4-NET axis as a critical driver of SLE renal phenotypes.

NADPH Oxidase-Derived •O_2_^−^: NE/MPO Pathway Activation and Arthralgia/Synovitis

The NOX2 in neutrophils produces •O_2_^−^, which is rapidly converted to H_2_O_2_ by SOD [137,140]. Unlike mtROS, •O_2_^−^/H_2_O_2_ primarily activates the NE/MPO pathway of NET formation: H_2_O_2_ triggers the release of NE and MPO from azurophilic granules, which then translocate to the nucleus to degrade histones and promote chromatin decondensation [104,137]. NETs generated via this pathway are enriched with oxidized genomic DNA and MPO, which activate the cGAS-STING pathway in synovial macrophages—inducing secretion of IL-17 and TNF-α [148]. Clinically, patients with SLE with active arthralgia exhibit higher NOX2 activity in peripheral neutrophils and increased circulating MPO-DNA complexes compared to patients without joint involvement [148]. Antioxidants targeting NADPH oxidase reduce •O_2_^−^ production, inhibit NE/MPO-mediated NETosis, and alleviate synovial inflammation in murine lupus models [149], highlighting this ROS-NET axis as a driver of SLE musculoskeletal phenotypes.

MPO-Derived HOCl: Histone Oxidation and Cutaneous Lupus Erythematosus (CLE)

MPO, activated by NADPH oxidase-derived H_2_O_2_, catalyzes the production of HOCl—a highly reactive oxidant with unique specificity for histone oxidation [150,151]. HOCl mediates the carbamylation of histone lysine residues, thereby disrupting histone–DNA interactions and accelerating chromatin decondensation and NET release [47,102]. NETs formed via HOCl-driven oxidation are enriched in carbamylated histones, which act as autoantigens for anti-histone antibodies [152]. These NET-ICs deposit in the dermal–epidermal junction, activating keratinocytes to secrete type I IFNs and chemokines—leading to the development of discoid lupus erythematosus or subacute cutaneous lupus erythematosus (SCLE) lesions [152]. In a cohort study of 52 CLE patients, skin biopsies showed colocalization of HOCl-modified histones, NETs, and anti-histone antibodies at lesional sites. At the same time, serum HOCl levels correlated with CLE disease activity [152]. Antioxidants that inhibit MPO reduce HOCl production and CLE lesion severity in murine models [153], confirming the role of the HOCl-NET axis in SLE cutaneous phenotypes.

RNS: NET Degradation Impairment and Thrombotic SLE

In addition to ROS, RNS contributes to NET-related SLE phenotypes by inhibiting DNase-mediated NET clearance [116,127]. ONOO^−^ is generated by the reaction of NO with •O_2_^−^; in SLE, excessive ONOO^−^ oxidizes the active site of DNase1 and DNase1L3, reducing their ability to degrade NET DNA [103]. Accumulated NETs then act as a scaffold for platelet activation: ONOO^−^-modified NET histones bind platelet TLR2/TLR4, while NE activates platelet PAR4, thus triggering platelet aggregation and thrombus formation [117,118]. Clinically, patients with SLE with APS exhibit higher plasma ONOO^−^ levels and lower DNase1 activity, with circulating ONOO^−^-NET complexes correlating with increased risk of deep vein thrombosis (DVT) [103]. Antioxidants that scavenge RNS reduce ONOO^−^ levels, restore DNase activity, and decrease thrombotic events in murine lupus-APS models [154], linking RNS-NET clearance impairment to SLE thrombotic phenotypes.

## 5. Potential Applications of Antioxidants in NETs and SLE

### 5.1. Classification and Mechanisms of Action of Antioxidants

Antioxidants are a diverse group of compounds that play a crucial role in mitigating oxidative damage and maintaining cellular redox homeostasis [155]. They can be broadly classified into three categories—enzymatic antioxidants, non-enzymatic antioxidants, and natural antioxidants—each with distinct mechanisms of action [156].

Enzymatic antioxidants, such as SOD, catalase, and glutathione peroxidase (GPx), are integral to the body’s defense against ROS [157]. SOD catalyzes the dismutation of superoxide radicals into oxygen and H_2_O_2_, thereby reducing their cytotoxic effects. Catalase further decomposes H_2_O_2_ into water and oxygen, while GPx uses reduced GSH to convert H_2_O_2_ and organic peroxides into water and the corresponding alcohols, respectively [158]. These enzymes work synergistically to neutralize ROS and protect cells from oxidative damage [158,159].

Non-enzymatic antioxidants include vitamins C and E, and GSH [160]. Vitamin C, a water-soluble antioxidant, donates electrons to neutralize free radicals such as the •OH and •O_2_^−^, thus preventing oxidative damage to cellular components [161]. Vitamin E, a lipid-soluble antioxidant, is particularly effective in intercepting peroxyl radicals in cell membranes and lipoproteins, thereby interrupting the propagation of lipid peroxidation [162]. GSH, a tripeptide thiol, maintains the cellular redox balance by cycling between its reduced and oxidized forms and serves as a substrate for GPx in the reduction of peroxides [163].

Natural plant- and other-source antioxidants encompass a wide array of compounds with potent antioxidant properties. Resveratrol, a polyphenol found in grapes and red wine, exhibits antioxidant, anti-inflammatory, and anticancer activities by scavenging free radicals, inhibiting oxidative enzymes, and modulating intracellular signaling pathways [164]. Curcumin, another polyphenol from turmeric, possesses potent antioxidant properties and can suppress inflammation and apoptosis through multiple mechanisms [165]. Green tea polyphenols, primarily composed of catechins, exert antioxidant effects by directly quenching free radicals, inhibiting oxidative enzyme activities, and modulating the cellular antioxidant defense system [166].

In summary, antioxidants, through their diverse mechanisms of action, collectively neutralize ROS and RNS, thereby safeguarding cells from oxidative damage [167]. Enzymatic antioxidants catalyze the decomposition of ROS, non-enzymatic antioxidants directly scavenge free radicals, and natural antioxidants modulate cellular redox balance through various pathways [168]. The synergistic action of these antioxidants is essential for maintaining cellular integrity and preventing diseases associated with oxidative stress.

### 5.2. Effects of Antioxidants on NET Formation

NETs are a crucial component of the innate immune response, enabling neutrophils to capture and neutralize pathogens [169]. However, excessive or inappropriate NET formation has been implicated in various inflammatory and autoimmune diseases, including SLE, making NETs a potential therapeutic target [170]. Antioxidants have emerged as a promising strategy to modulate NET formation through multiple mechanisms.

Direct scavenging of ROS is a primary mechanism by which antioxidants inhibit NET formation. ROS, particularly HOCl produced by MPO, play a pivotal role in triggering NETosis [171]. Studies have shown that thiocyanate (SCN-) and selenocyanate (SeCN-) can effectively neutralize HOCl. This reduces NET release in neutrophils stimulated with PMA or bacterial peptides such as nigericin [150]. For instance, Hallberg et al. found differentiated PLB-985 cells and primary human neutrophils treated with SCN- had reduced NET release after PMA stimulation, confirming the effectiveness of direct ROS scavenging in regulating NET formation [150].

Inhibition of NADPH oxidase is another mechanism by which antioxidants can suppress NET formation [172]. NADPH oxidase is essential for ROS production, which is necessary for NET release [173]. The NADPH oxidase inhibitor diphenylene iodonium (DPI) has been shown to reduce NET formation effectively in multiple studies. For example, in a survey by Shirakawa et al. [173], Hydrogen gas (H_2_) inhibited PMA-induced NET formation in human neutrophils by neutralizing HOCl, a potent oxidant generated by NADPH oxidase, thereby suppressing DNA damage and NET release. This suggests that targeting NADPH oxidase activity is a viable strategy for controlling NET formation.

Antioxidants can also inhibit the activity of MPO and elastases, enzymes involved in NET formation [174]. MPO-derived ROS, such as HOCl, contribute to the release of DNA and histones, key components of NETs [175]. Inhibiting MPO can reduce ROS formation and, consequently, decrease NET release [176]. SeCN-, for example, has been shown to inhibit MPO activity, thereby reducing NET formation in neutrophils [150]. This highlights the importance of targeting MPO in modulating NET formation.

**Figure 2:**
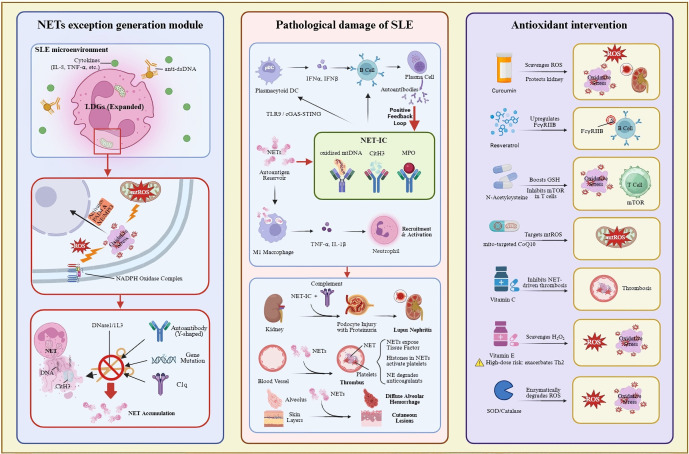
Schematic diagram of NETs-related pathological pathways in SLE and targeted intervention of antioxidants. Abnormal NET formation driven by the SLE microenvironment (e.g., LDGs, immune complexes) and oxidative stress leads to NET accumulation, which induces tissue damage (e.g., kidney, blood vessels) and inflammatory amplification. Antioxidants inhibit NET formation and alleviate SLE pathology through multiple mechanisms, including ROS scavenging, inhibition of NADPH oxidase, and regulation of mitochondrial function.

In conclusion, antioxidants offer a multifaceted approach to inhibit NET formation by directly scavenging ROS, inhibiting NADPH oxidase activity, and targeting MPO and elastase [150,172,173,176]. These strategies have shown promises in reducing NET formation in vitro and in vivo, suggesting that antioxidants could be a valuable therapeutic tool for managing NET-related diseases. Future research should focus on optimizing antioxidant formulations and exploring their efficacy in clinical settings to harness their potential in treating inflammatory and autoimmune conditions. The regulatory mechanism of the above-mentioned antioxidants on NET formation can be further clarified through the visualization path (Figure 2).

### 5.3. Potential Applications of Antioxidants in SLE Therapy

#### 5.3.1. Preclinical Studies

SLE is a complex autoimmune disease characterized by widespread inflammation, organ involvement, and the production of autoantibodies [177]. Oxidative stress, resulting from an imbalance between ROS production and the body’s antioxidant defense, has been implicated in the pathogenesis of SLE [151]. This imbalance is believed to contribute to NET formation, which is associated with increased inflammation and tissue damage in patients with SLE [178]. Accumulating evidence from both in vitro and animal model studies has demonstrated that antioxidants can significantly reduce NET formation and alleviate the pathological symptoms of SLE.

Curcumin, a natural polyphenolic compound with anti-inflammatory and antioxidant properties. In MRL/lpr mice SLE model, it has been shown to significantly reduce proteinuria and renal inflammation [179]. This effect is attributed to its ability to inhibit activation of the NLR family pyrin domain-containing 3 (NLRP3) inflammasome, a key player in the pathogenesis of lupus nephritis [180]. Additionally, Yang et al. [149] demonstrated curcumin’s efficacy in alleviating lupus nephritis in two mouse models (MRL/lpr and R848-treated). The compound works by regulating the PI3K/AKT/NF-κB signaling pathway, thereby inhibiting neutrophil migration and the release of inflammatory factors.

Recent evidence suggests that resveratrol ameliorates immune disorders by inhibiting the overactivation of immune cells, making it a promising therapeutic option for SLE. Wang et al. [181] demonstrated that resveratrol could suppress B cell proliferation and induce apoptosis in CD4+ T cells, thereby reducing autoantibody production and alleviating lupus-like symptoms. Additionally, resveratrol upregulated FcγRIIB expression in B cells via NF-κB activation, leading to reduced B cell numbers, decreased serum autoantibody levels, and improved lupus nephritis in MRL/lpr mice [182]. Similarly, the combination of resveratrol and piperine has been reported to mitigate renal, hepatic, and pulmonary manifestations in a pristane-induced SLE mouse model. Research by Pannu et al. [183] proved that resveratrol combined with piperine mitigated renal manifestations, limited NET-mediated tissue damage, alleviated oxidative stress, reduced inflammatory cytokines and lipogranuloma formation, but failed to abrogate autoantibody production or improve spleen/skin manifestations in a pristane-induced SLE murine model. They also demonstrated that resveratrol, alone and in combination with piperine, effectively mitigated oxidative stress and inflammation in a pristane-induced lupus-like murine model, reducing renal pathology and ICs. However, neither treatment regulated autoantibody formation, nor did combining resveratrol with piperine enhance its efficacy compared with resveratrol alone [153].

Vitamins C and E, owing to their antioxidant properties, are advantageous in the treatment of SLE. A recent study showed that vitamin C contributed to treating SLE by acting as a hypoxia-inducible factor-1α (HIF-1α) inhibitor, which down-regulated the expression of the stress-response protein, suppressed autophagy induction in neutrophils, reduced the release of NETs decorated with TF and IL-17A, and thereby mitigated thromboinflammation and fibrosis associated with end-organ injury in SLE [148]. Mohammed et al. [184] found that vitamin C inhibits NETosis in polymorphonuclear neutrophils from healthy mice. In a hydralazine-induced lupus mouse model, vitamin E at a higher dose (50 mg/kg) significantly reduced lymphocyte hydrogen peroxide radicals compared to a lower dose (25 mg/kg), indicating a dose-dependent scavenging potential and suggesting it may be a promising therapeutic agent for SLE [152]. Another study demonstrated that low-dose vitamin E supplementation (250 mg/kg) extended lifespan, whereas high-dose supplementation (500 mg/kg) shortened it. High doses increased anti-dsDNA and anticardiolipin antibody levels, suppressed IL-2, and boosted IL-4 and IL-10—suggesting that high vitamin E intake may exacerbate Th2-driven autoimmune diseases such as lupus in MRL/lpr mice [185].

Other antioxidants, such as coenzyme Q10 and NAC, have also been shown to inhibit NET formation by scavenging ROS and reducing oxidative stress. Blanco et al. [147] demonstrated that coenzyme Q10 analog Idebenone not only downregulated NET formation in neutrophils and inhibited NET formation in lupus-prone mice, but also enhanced mitochondrial metabolism and ATP production, improved endothelium-dependent vasorelaxation, and reduced lipid peroxidation, indicating its potential as a therapeutic agent for SLE. In a parallel study, Fortner et al. [146] explored the therapeutic potential of the mitochondrial-targeted coenzyme Q10 (MitoQ) in the manifestations of lupus using MRL/lpr mice. In this study, lupus-prone MRL/lpr mice were treated with MitoQ (200 µM) for eleven weeks. After treatment, the mice exhibited reduced NETosis and ROS production, decreased serum IFN levels, and diminished IC formation in the kidneys. NAC has been demonstrated to decrease the expression of pro-inflammatory cytokines and chemokines, thereby mitigating the inflammatory response in SLE. In addition, in vitro experiments have indicated that NAC effectively reduced NET formation and oxidative stress in neutrophils by enhancing antioxidant capacity and decreasing ROS and lipid peroxidation levels [186].

#### 5.3.2. Clinical Trials

Clinical trials evaluating the effects of antioxidants in the treatment of SLE have yielded promising results. Preliminary small-scale trials (*n* = 24–70) have shown that curcumin (1000–1500 mg/day) improves select clinical and laboratory outcomes in patients with SLE—including reduced proteinuria, anti-dsDNA levels, and IL-6 concentrations—with no severe adverse events. For example, a randomized, placebo-controlled study investigated the effects of oral curcumin supplementation in 24 patients with relapsing or refractory biopsy-proven lupus nephritis; results showed that the trial group had significant reductions in proteinuria), systolic blood pressure, and hematuria, with no adverse effects related to turmeric, leading to the conclusion that short-term turmeric supplementation can be a safe adjuvant therapy for patients with relapsing or refractory lupus nephritis [187]. In another randomized, triple-blinded, placebo-controlled trial, curcumin supplementation for 10 weeks significantly reduced anti-dsDNA and IL-6 levels in patients with SLE, demonstrating its potential as an effective and safe adjuvant therapy to ameliorate autoimmune activity and inflammation [188]. However, a double-blind randomized controlled trial conducted in Saiful Anwar Hospital demonstrated that supplementing with curcuma (20 mg/day) in addition to vitamin D_3_ for three months did not significantly affect SLE Disease Activity Index (SLEDAI) scores, IL-6 levels, or transforming growth factor-β1 (TGF-β1) levels in patients with SLE with hypovitamin D compared to vitamin D_3_ alone [189].

Several ongoing clinical trials are further investigating the potential benefits of NAC in SLE. For instance, a randomized, double-blind clinical trial treated patients with SLE with NAC (1800 mg/day) for 3 months. It found a significant reduction in disease activity scores, measured by SLEDAI and the British Isles Lupus Assessment Group (BILAG) criteria [190]. Another study by Li et al. [191] reported two cases of early-stage lupus nephritis treated with NAC. The patients received 1200 mg/day of NAC for 3 months, resulting in significant improvements in GSH levels, reductions in lipid peroxidation biomarkers, and overall clinical improvement. These findings suggest that NAC may be effective in modulating oxidative stress and improving disease outcomes in early-stage lupus nephritis. In addition to its antioxidant properties, NAC has been shown to block the mammalian target of rapamycin (mTOR) signaling pathway in T cells, which is implicated in SLE pathogenesis. Lai et al. [192] conducted a randomized, double-blind, placebo-controlled trial involving 36 patients with SLE, demonstrating that NAC significantly reduced disease activity and fatigue by inhibiting mTOR. The study also revealed that NAC increased mitochondrial membrane potential and enhanced apoptosis in T cells, contributing to the overall therapeutic effect. Furthermore, comprehensive metabolome analyses by Perl et al. [193] identified significant changes in patients with SLE’ metabolomes, particularly in the pentose phosphate pathway. NAC treatment reduced kynurenine accumulation, a metabolite that activates mTOR, providing a metabolic basis for its therapeutic efficacy in SLE. In the context of neuropsychiatric manifestations, Garcia et al. [194] reported that NAC significantly improved attention deficit and hyperactivity disorder (ADHD) symptoms in patients with SLE. The study used the ADHD Self-Report Scale (ASRS) to assess cognitive and hyperactivity symptoms and found that NAC treatment led to significant reductions in ASRS scores, suggesting its potential to address cognitive dysfunction in SLE. Finally, Doherty et al. [154] investigated the effects of NAC on mitochondrial function in patients with SLE. The study found that NAC reduced oxygen consumption through mitochondrial electron transport chain (ETC) complex I, thereby decreasing oxidative stress. These findings further support NAC’s role in modulating mitochondrial dysfunction and oxidative stress in SLE. In conclusion, the cumulative evidence from these studies indicates that NAC is a promising therapeutic agent for SLE, offering multiple benefits through its antioxidant properties, modulation of mitochondrial function, and inhibition of the mTOR signaling pathway.

In addition to these trials, several studies have investigated the potential synergistic effects of vitamin C and vitamin E with conventional SLE therapies. Comstock et al. [195] conducted a prospective case–control study to investigate serum concentrations of vitamin E, β-carotene, and retinol before the diagnosis of rheumatoid arthritis and SLE. The study found that individuals who later developed SLE had lower serum concentrations of these antioxidants than matched controls, suggesting that low antioxidant status may be a risk factor for SLE. This finding was supported by Bae et al. [196], who compared plasma antioxidant/oxidant status and dietary nutrient intake in patients with SLE and healthy controls. They found that patients with SLE had significantly lower plasma SOD and GPx activities, higher MDA levels, and lower dietary intake of vitamin A, β-carotene, and vitamin C. These results indicated that patients with SLE have impaired antioxidant status and reduced dietary intake of antioxidants.

Maeshima et al. [197] conducted a preliminary study to evaluate the efficacy of vitamin E in reducing oxidative DNA damage and autoantibody production in patients with SLE. The study measured urinary 8-hydroxydeoxyguanosine (8-OHdG) as an indicator of oxidative DNA damage and anti-dsDNA antibody levels as a predictor of disease activity. While urinary 8-OHdG levels did not differ significantly between patients receiving vitamin E and those not receiving vitamin E, anti-ds DNA antibody titers were significantly lower in the vitamin E group. This suggests that vitamin E may suppress autoantibody production in SLE, potentially through mechanisms independent of its antioxidant activity.

In conclusion, both preclinical and clinical studies have highlighted the potential therapeutic benefits of antioxidants in SLE (Table 1). Further research is warranted to elucidate the precise mechanisms by which antioxidants exert their effects and to determine the optimal dosing and combinations for clinical use. Future studies should also focus on long-term outcomes and the potential for antioxidants to modify disease progression in SLE. Additionally, research should explore the synergistic effects of antioxidants with other therapeutic agents to develop more effective and safer treatment strategies for patients with SLE.

### 5.4. Barriers to Antioxidants as Standard Adjunct Therapy in SLE

Despite promising preclinical and preliminary clinical evidence supporting the use of antioxidants as adjuncts in SLE, they have not yet been integrated into standard clinical practice. This gap arises from multifaceted challenges spanning clinical evidence quality, pharmacokinetic limitations, safety uncertainties, and lack of alignment with existing treatment frameworks. These challenges are detailed below:

Limited Rigor of Clinical Evidence: Most clinical trials of antioxidants in SLE are small-scale and short-term [187,190,191]. For example, curcumin trials [187,188] demonstrated reduced proteinuria and anti-dsDNA levels, but they lacked long-term (≥12 months) data on disease modification. Additionally, trial outcomes are inconsistent: while curcumin (1500 mg/day) improved renal parameters in relapsing lupus nephritis [187], a combination of curcumin and vitamin D3 did not reduce SLEDAI scores in patients with hypovitaminosis D and SLE [189]. Such inconsistencies weaken the evidence grade, as standard therapies require reproducible efficacy across large, diverse cohorts per European League Against Rheumatism/American College of Rheumatology clinical trial guidelines.

Unresolved Dosage and Safety Profiles: Antioxidant efficacy and safety are highly dose-dependent, but optimal dosing for SLE remains undefined. For instance, low-dose vitamin E (25–250 mg/kg) extended lifespan and reduced oxidative damage in MRL/lpr mice [185], while high-dose vitamin E (≥500 mg/kg) increased anti-dsDNA antibodies and exacerbated Th2-driven autoimmunity [185]. In humans, clinical trials of NAC used doses ranging from 1200 to 1800 mg/day [190,191]. Still, no dose-escalation studies have identified the minimum effective dose or the maximum tolerated dose for SLE. Furthermore, long-term safety data are absent, which is critical for patients with SLE requiring lifelong adjunct therapy.

Poor Pharmacokinetics and Bioavailability: Many antioxidants with preclinical efficacy exhibit suboptimal pharmacokinetics and bioavailability. Curcumin, a well-studied polyphenol, has extremely low oral bioavailability due to rapid first-pass metabolism in the liver and intestinal degradation [179]. While piperine is used to enhance curcumin absorption [183], clinical trials of curcumin–piperine combinations in SLE [183] showed no synergistic improvement in autoantibody reduction compared to curcumin alone, suggesting that bioavailability enhancement may not fully translate to clinical benefit. Similarly, resveratrol exhibits a short plasma half-life and extensive conjugation, limiting its tissue penetration to target organs [181].

Lack of Evaluation in Combination with Standard SLE Therapies: Current antioxidant trials primarily evaluate monotherapy or adjunctive use with low-dose corticosteroids, not frontline SLE treatments. For example, NAC trials [190,192] excluded patients on high-dose immunosuppressants, leaving uncertainty about potential drug–drug interactions. Without data on combination therapy, clinicians are reluctant to adopt antioxidants, as SLE management relies on multitargeted regimens to control complex pathogenesis.

Absence of Guidelines and Regulatory Endorsement: Most SLE treatment guidelines do not mention antioxidants, as the evidence is deemed “insufficient” for grade B/C recommendations. Regulatory agencies have not approved any antioxidant for adjunct therapy in SLE, partly due to the lack of phase III trials demonstrating patient-relevant outcomes beyond surrogate markers. This regulatory gap further hinders clinical adoption, as clinicians prioritize guideline-endorsed interventions.

**Table 1:** Characteristics of 21 Studies on Applications of Antioxidants in SLE Therapy.

Antioxidants	Category	Sample Model	Dosage	Results	Reference
Curcumin	preclinical study	female MRL/lpr mice	200 mg/kg/day	Curcumin effectively reduces proteinuria, renal inflammation, serum anti-dsDNA levels, and spleen size, and inhibits NLRP3 inflammasome activation both in vivo and in vitro.	Zhao et al., 2019 [180]
	preclinical study	MRL/lpr mice and R848-treated mice	50 mg/kg/day	Curcumin effectively reduces renal inflammation in lupus mouse models by inhibiting neutrophil migration and inflammatory factor release via the PI3K/AKT/NF-κB signaling pathway.	Yang et al., 2024 [149]
Resveratrol	preclinical study	pristane-induced lupus mice	50 mg/kg/day; 75 mg/kg/day	Resveratrol significantly mitigates proteinuria, kidney immunoglobulin deposition, and glomerulonephritis in pristane-induced lupus mice, which also suppresses CD4^+^ T cell activation and proliferation, induces CD4^+^ T cell apoptosis, and inhibits B cell antibody production and proliferation in vitro.	Wang et al., 2014 [181]
	preclinical study	MRL/lpr mice	20 mg /kg/day	Resveratrol alleviates lupus symptoms in MRL/lpr mice by enhancing FcγRIIB expression in B cells via Sirt1 activation, reducing plasma cells and autoantibodies, and improving nephritis and survival.	Jhou et al., 2017 [182]
	preclinical study	pristane-induced lupus mice	25 mg/kg/day; 50 mg/kg/day	In a pristane-induced SLE murine model, low-dose resveratrol combined with piperine and high-dose resveratrol reduced renal immunoglobulin deposition, hepatic lipogranuloma formation, and pulmonary inflammation, reduced oxidative stress, and improved lupus symptoms, but did not affect autoantibody formation or spleen/skin manifestations.	Pannu et al., 2020 [183]
	preclinical study	pristane-induced lupus mice	25 mg/kg/day	Resveratrol alone and in combination with piperine effectively mitigated oxidative stress and inflammation, improved renal function, and reduced histopathological manifestations in a pristane-induced lupus murine model. Still, neither treatment regulated autoantibody formation, and piperine did not enhance resveratrol’s efficacy.	Pannu et al., 2020 [153]
Vitamins C	preclinical study	peripheral blood neutrophils isolated from patients with active SLE	10 mM/day	Vitamin C inhibits NETosis in SLE neutrophils by targeting the REDD1/autophagy/NET axis, reducing thromboinflammation and fibrosis.	Frangou et al., 2018 [148]
	preclinical study	Gulo-/- mice	200 mg/kg/day	Vitamin C reduces NETosis in sepsis by attenuating ER stress, autophagy, histone citrullination, and NFκB activation.	Mohammed et al., 2013 [184]
Vitamins E	preclinical study	hydralazine-induced lupus mice	25 mg/kg/day; 50 mg/kg/day	Vitamin E, particularly at a higher dose (50 mg/kg), shows potential in reducing lymphocyte hydrogen peroxide radicals in a hydralazine-induced lupus mouse model.	Githaiga et al., 2023 [152]
	preclinical study	MRL/lymphoproliferative lpr female mice	50 mg/kg/day; 250 mg/kg/day; 375 mg/kg/day; 500 mg/kg/day	Low-dose vitamin E extends lifespan in MRL/lpr mice, whereas high-dose vitamin E increases Th2 cytokine production and autoantibody levels, potentially worsening Th2-driven autoimmune diseases such as SLE.	Hsieh et al., 2005 [185]
Coenzyme Q10	preclinical study	MRL/lpr mice	1 mg/kg; 1.5 mg/kg	Coenzyme Q10 significantly reduces mortality, attenuates disease features, and improves mitochondrial function, renal function, and inflammation in lupus mouse models, supporting its potential therapeutic role in SLE.	Blanco et al., 2020 [147]
	preclinical study	MRL/lpr mice	MitoQ (200 µM) in drinking water/day	MitoQ reduces neutrophil ROS and NET formation, MAVS oligomerisation, and serum IFN-I in lupus-prone MRL-lpr mice, highlighting the potential of targeting mROS as an adjunct therapy for lupus.	Fortner et al., 2020 [146]
Curcumin	clinical trial	24 patients with relapsing or refractory biopsy-proven lupus nephritis	1500 mg/day	Short-term curcumin supplementation significantly reduced proteinuria, hematuria, and systolic blood pressure in patients with relapsing or refractory lupus nephritis.	Khajehdehi et al., 2012 [187]
	clinical trial	70 SLE patients	1000 mg/day	Curcumin supplementation significantly reduced anti-dsDNA and IL-6 levels in SLE patients, with no significant changes in other variables.	Sedighi et al., 2024 [188]
	clinical trial	SLE active (SLEDAI > 3) with levels of 25(OH)D3 ≤ 30 ng/mL SLE patients	1200 IU/day	Curcumin combined with vitamin D_3_ showed no significant effects on SLEDAI and serum levels of IL-6 and TGF-β1 in SLE patients with low vitamin D levels. However, decreased IL-6 levels were positively correlated with reductions in SLEDAI.	Singgih et al., 2017 [189]
N-acetylcysteine	clinical trial	80 SLE patients	1800 mg/day	NAC (1800 mg/day) significantly reduced SLE disease activity and complications, as evidenced by lower BILAG and SLEDAI scores and improved CH50 levels after 3 months, with no adverse events reported.	Abbasifard et al., 2023 [190]
	clinical trial	female SLE patients	1200 mg/day	NAC treatment in early-stage lupus nephritis led to increased GSH levels, decreased levels of lipid peroxidation biomarkers, and significant improvements in routine blood counts, 24-h urine protein, erythrocyte sedimentation rate, and SLEDAI.	Li et al., 2015 [191]
	clinical trial	36 SLE patients	1.2 mg/day; 2.4 mg/day; 4.8 mg/day	NAC at 2.4 and 4.8 mg/day significantly reduced SLE activity scores and fatigue levels, while improving mitochondrial function, reducing mTOR activity, enhancing apoptosis, and decreasing anti-dsDNA antibody production in SLE patients.	Lai et al., 2012 [192]
	clinical trial	49 SLE patients and 46 healthy control subjects	2.4 mg/day; 4.8 mg/day	NAC treatment at dosages of 2.4 and 4.8 mg/day significantly reduced ADHD symptoms in SLE patients, as indicated by lower ASRS total and part A scores, demonstrating its efficacy in improving cognitive and inattentive aspects of ADHD in this patient group.	Garcia et al., 2013 [194]
	clinical trial	69 SLE patients and 37 healthy donors	3 mg/day	NAC treatment effectively reduced oxygen consumption via mitochondrial ETC complex I and H_2_O_2_ levels in peripheral blood lymphocytes from SLE patients, indicating its potential therapeutic efficacy in reducing oxidative stress.	Doherty et al., 2014 [154]
Vitamins E	clinical trial	12 SLE patients	150–300 mg/day	Vitamin E can suppress autoantibody production in SLE patients, as indicated by lower anti-dsDNA antibody titers, independent of its antioxidant activity.	Maeshima et al., 2007 [197]

## 6. Conclusions

NETs are significant pathogenic factors in SLE, and their abnormal formation, accumulation, and oxidative stress are closely linked. Under normal conditions, NETs help capture pathogens. In SLE, however, pro-inflammatory LDGs spontaneously form NETs. These NETs release autoantigens, which then form ICs with antibodies like anti-dsDNA.

These NET-ICs have two key pathogenic effects: to stimulate pDCs to secrete type I IFNs, granular proteins, and NE; and to promote the deposition of IFNs in target organs (e.g., kidneys, skin), resulting in tissue damage. Furthermore, patients with SLE exhibit impaired NET clearance. This defect arises from reduced DNase1 and DNase1L3 activity or the presence of autoantibodies against these nucleases, creating a vicious cycle of autoimmune activation.

Oxidative stress plays a pivotal role in mediating key steps of NET formation, such as cytoskeleton disruption and chromatin decondensation. Driven by the NADPH oxidase complex and mitochondria, ROS not only promote NET formation but also damage cellular components—exacerbating SLE pathogenesis.

Antioxidants, including enzymatic, non-enzymatic, and naturally derived antioxidants, can help inhibit abnormal NET formation by scavenging ROS, suppressing NADPH oxidase activity, or reducing MPO activity. Preclinical studies have shown that curcumin, resveratrol, and mitochondria-targeted antioxidants can alleviate symptoms such as proteinuria and renal inflammation in mouse models of SLE. Clinical studies have further confirmed that curcumin, NAC, and other antioxidants can reduce SLE disease activity and relieve related symptoms, while low-dose vitamin E has also shown potential to reduce oxidative DNA damage. These findings support antioxidants as safe adjunctive therapies for SLE.

Future research should prioritize three areas: (1) defining the functional heterogeneity of NET subtypes and their distinct roles in SLE; (2) addressing antioxidant limitations via targeted delivery systems; and (3) developing combination therapies that co-target NETs, oxidative stress, and type I IFN signaling. Challenges in developing antioxidants, including low bioavailability, limited tissue targeting, and potential side effects at high doses, need to be addressed. Additionally, therapies that combine approaches against NETs, oxidative stress, and other drivers of SLE should be developed.

In summary, NETs are central pathogenic mediators in SLE, with oxidative stress as a key driver of their dysregulation. Antioxidants—when optimized for dose, bioavailability, and patient-specific factors—offer a promising adjuvant strategy to reduce NET formation, alleviate symptoms like proteinuria, and potentially modify SLE progression. However, successful translation requires addressing formulation challenges, conducting long-term safety trials, and tailoring therapies to the molecular heterogeneity of SLE.

## Data Availability

No new data were created or analyzed in this study. Data sharing is not applicable to this article.

## Abbreviations

The following abbreviations are used in this manuscript:

•O_2_−	superoxide anion radicals
•OH	hydroxyl radicals
4-HNE	4-hydroxynonenal
8-OHdG	8-hydroxydeoxyguanosine
ABCs	age-associated B cells
ADHD	attention deficit hyperactivity disorder
ANA	antinuclear antibodies
anti-dsDNA	anti-double-stranded DNA
anti-Sm	anti-Smith
anti-β2-GPI	anti-β2-glycoprotein I
APS	antiphospholipid syndrome
ASRS	ADHD Self-Report Scale
BCRs	B cell receptors
BILAG	British Isles Lupus Assessment Group
C1q	complement component 1q
Ca^2+^	calcium
cDCs	conventional dendritic cells
CitH3	citrullinated histone H3
CLE	cutaneous lupus erythematosus
DAH	diffuse alveolar hemorrhage
DAMP	damage-associated molecular pattern
DCs	dendritic cells
DNase1L3	Deoxyribonuclease 1-like 3
DPI	diphenylene iodonium
DVT	deep vein thrombosis
EndMT	endothelial-to-mesenchymal transition
FcγRs	Fcγ receptors
GPx	glutathione peroxidase
GSDMD	gasdermin D
GSH	glutathione
Gsr	glutathione reductase
H_2_	hydrogen gas
H_2_O_2_	hydrogen peroxide
HDNs	normal-density neutrophils
HIF-1α	hypoxia-inducible factor-1α
HMGB1	high-mobility group box 1
HOCl	hypochlorous acid
ICs	immune complexes
IFNs	interferons
IL-17	interleukin-17
IL-1β	interleukin-1β
IL-21	interleukin-21
IL-33	interleukin-33
LDGs	low-density granulocytes
MAPK	mitogen-activated protein kinase
MDA	malondialdehyde
MitoQ	mitochondrial-targeted coenzyme Q10
MMPs	matrix metalloproteinases
MPO	myeloperoxidase
mtDNA	mitochondrial DNA
mTOR	mammalian target of rapamycin
mtROS	mitochondrial reactive oxygen species
NAC	N-acetylcysteine
NADPH	nicotinamide adenine dinucleotide phosphate
NE	neutrophil elastase
NETs	neutrophil extracellular traps
NF-κB	nuclear factor-kappa B
NLRP3	NLR family pyrin domain containing 3
NO	nitric oxide
ONOO−	peroxynitrite
PAD4	peptidylarginine deiminase 4
PAR4	protease-activated receptor 4
pDCs	plasmacytoid dendritic cells
PKC	protein kinase C
PMA	phorbol 12-myristate 13-acetate
PRKCD	protein kinase C delta
PRRs	pattern recognition receptors
REDD1	regulated in development and DNA damage response 1
RNS	reactive nitrogen species
ROS	reactive oxygen species
SCLE	subacute cutaneous lupus erythematosus
SCN-	thiocyanate
SeCN-	selenocyanate
SLE	systemic lupus erythematosus
SLEDAI	SLE Disease Activity Index
SOD	superoxide dismutase
Tfh	T follicular helper cells
TFPI	tissue factor pathway inhibitor
TGF-β1	transforming growth factor-β1
TLR7	Toll-like receptor 7
TLR9	Toll-like receptor 9
TNF-α	tumor necrosis factor-α
Tph	T peripheral helper cells
